# A Randomized Controlled Trial Comparing Ultrasonography-Guided Needle Aspiration and Surgical Drainage for the Management of Breast Abscess

**DOI:** 10.7759/cureus.50956

**Published:** 2023-12-22

**Authors:** Bishal Pal, Oseen Shaikh, Chellappa Vijayakumar, Sagar Prakash, Gopal Balasubramanian, Uday Kumbhar

**Affiliations:** 1 Surgery, Jawaharlal Institute of Postgraduate Medical Education and Research, Puducherry, IND

**Keywords:** breastfeeding, needle aspiration, ultrasonography, incision and drainage, breast abscess

## Abstract

Background

A breast abscess is a localized collection of inflammatory exudate in breast tissue. It is more common in lactating women. Treatment of the breast abscess is usually by incision and drainage, which is accompanied by extensive trauma, lengthy surgical incisions, psychological distress, surgical scar, and discomfort during dressing changes. Recent research has shown that ultrasonography-guided needle aspiration is an alternative to incision and drainage for breast abscess treatment, has superior cosmetic outcomes, and has fewer costs. However, there are no clear guidelines for the same. The primary objective of this study was to assess and contrast the outcomes and efficacy of two approaches in the treatment of breast abscesses: ultrasonography-guided needle aspiration and conventional incision and drainage.

Methods

This was a single-center, prospective, randomized, controlled, non-inferiority trial. Patients with breast abscesses were randomized either to receive needle aspiration or incision drainage. Patients with lactational breast abscesses were encouraged to breastfeed from either breast. The antibiotic was given for 10 days to all the patients. The study's main focus was on the primary outcome, which was the failure rate. Additionally, several secondary outcomes were examined, including postoperative pain, time required for healing, presence of residual abscess or recurrence, formation of fistulas, and the assessment of scar appearance.

Results

A total of 44 patients were randomized to 23 and 21 in each arm. There was no statistical difference in treatment failure (p-value=0.862), fistula formation (p-value=1.00), and recurrence (p-value=1.00). There was a significant statistical difference in healing period (p-value <0.001), scar formation (p-value <0.0001), continuation of breastfeeding (p-value=0.005), and clinical resolution (p-value=0.002). There was a significant reduction in post-intervention pain in the needle aspiration group than in the other group (p-value <0.001).

Conclusion

This study showed a significant difference in postoperative pain, healing time, the continuation of breastfeeding, and scar formation in the needle aspiration group, favouring minimally invasive treatment for breast abscess. However, due to the small sample size, we could not conclude that ultrasonography-guided needle aspiration of the breast abscess is non-inferior to the conventional incision and drainage.

## Introduction

A breast abscess (BA) is a confined accumulation of inflammatory exudates within breast tissue. Duct blockage, sore nipples, and infrequent breastfeeding are the leading risk factors for the development of BA. BA is more common in lactating mothers, and incidence ranges from 0.4 % to 11% [1]. It commonly develops during the first month of lactation and weaning [2]. Usually, there is a delay in the diagnosis due to social stigmata. Patients may present with painful lumps in the breast along with other signs of inflammation and fever. Patients may have fluctuant or non-fluctuant breast lump with or without axillary lymphadenopathy. Clinical examination, along with ultrasonography (USG), helps to arrive at the diagnosis [3].

Treatment of the BA at an early stage includes antibiotics, breast support garments, cold compression, and analgesics. Incision and drainage (ID) is the standard form of therapy for BA, which is accompanied by extensive trauma, lengthy surgical incisions, and discomfort during dressing changes [4]. Patients frequently experience excruciating agony and psychological distress due to surgical scars and breast distortion [5].

Recent research has shown USG-guided needle aspiration as a successful and more popular alternative to ID for BA treatment [6]. This method has been successfully performed and is linked to fewer recurrences, superior cosmetic outcomes, and lower costs [7]. Although it has been shown that these minimally invasive techniques can be effective and safe in treating breast abscesses with small diameters (<3 cm) or unilocular collections, there are still several potential drawbacks. These include a lower cure rate, higher recurrence risk than incisive surgical drainage, the need for multiple aspirations, and difficulty treating multiloculated or long-standing abscesses [8]. Therefore, surgical drainage is frequently necessary and suggested in situations like a failure of needle aspiration or catheter drainage, large abscesses with a minimum diameter of 5 cm or more, multiloculated abscesses, and chronic abscesses [8]. However, there needs to be clear guidelines and data regarding which method is better for drainage. This study aims to compare surgical drainage versus guided needle aspiration for BA. In addition, we also looked at recovery time and post-operative pain associated with surgical drainage versus USG-guided needle aspiration.

This paper was presented at the 82nd Annual Conference of the Associations of Surgeons of India (ASICON 2022, Mumbai, 21st to 24th December 2022) and received "Best Faculty Paper Award (Second prize)".

## Materials and methods

This study was a single-center, prospective randomized, controlled, non-inferiority trial done in a tertiary care hospital in south India from February 2020 to February 2022. The study received approval from the Postgraduate Research Monitoring Committee (PGRMC) and the Institute Ethics Committee (JIP/IEC/2021/018) at Jawaharlal Institute of Postgraduate Medical Education and Research. Additionally, the study was registered with the Clinical Trials Registry - India (CTRI) under the identifier CTRI/2020/05/025124.

All female patients with breast abscesses above 18 years of age presenting to the emergency or outpatient department (OPD) were included. Patients with spontaneous rupture of breast abscess, complicated breast abscess (ulceration, necrosis, multiloculated, suspicious malignancy), and immunocompromised individuals were excluded.

The sample size was calculated using nMaster version 2.02, considering the power of 80% and a level of significance as 5%, for declaring that the USG-guided needle aspiration is not inferior to the conventional ID at a 10% margin of non-inferiority (assuming that a larger proportion is desirable). Based on the previous literature review, a cure rate of 83% in the ID group and 95% in the USG-guided needle aspiration group, a total of 78 patients were included (39 in each group). 

**Figure 1:**
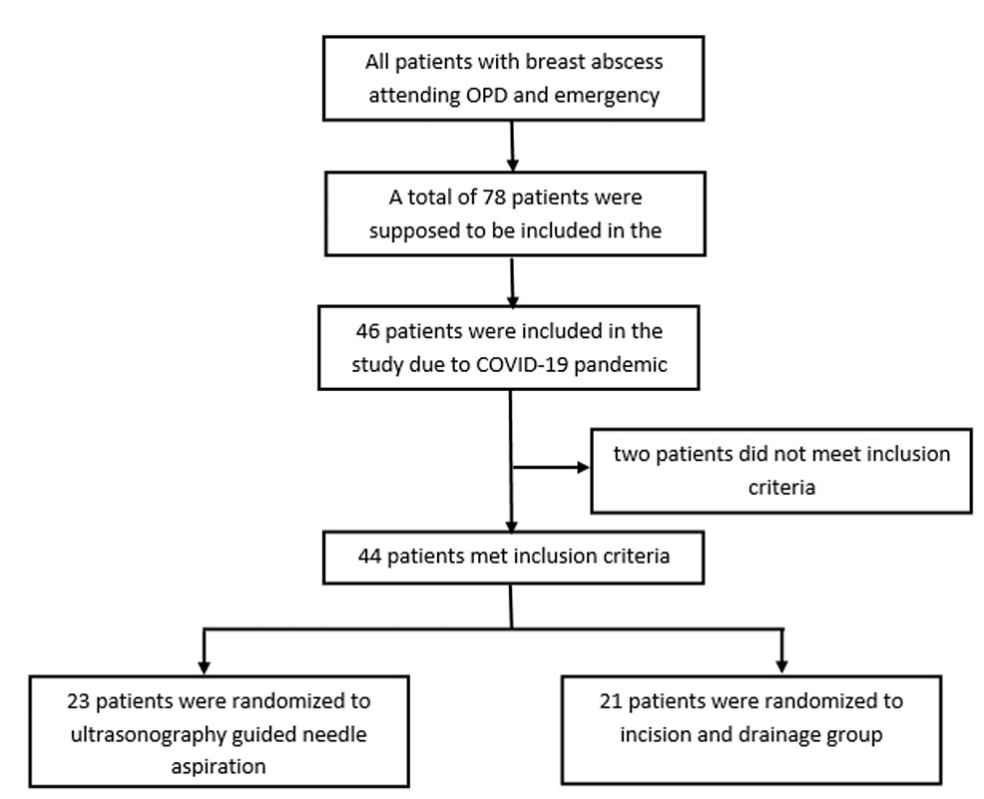
Consolidated Standards of Reporting Trials (CONSORT) diagram and allocation. OPD: Outpatient department; COVID-19: Coronavirus disease-19.

Categorical data were expressed as frequency or proportions or percentages. Quantitative data were expressed as mean with standard deviation (SD) or median with range. Categorical variables were compared using the Pearson chi-square test. Continuable variables were compared using an independent sample t-test. Significance was defined by p-values less than 0.05 using a two-tailed test. Data analysis was performed using Statistical Package for Social Sciences (SPSS) version 21.0 (IBM Corp., Armonk, NY, USA). Randomization was done by block randomization using allocation software and a concealed envelope. Group A patients underwent USG-guided needle aspiration, and in group B, patients underwent conventional ID (Figure 1).

Group A underwent USG-guided needle aspiration using an 18-gauge needle with a 20 ml syringe after administration of 2% lignocaine as a local anesthetic. The drained collection was sent for microbiological investigations. Repeat USG was carried out on the third and fifth day, and if there was a residual collection, re-aspiration was done twice. A clinical assessment was done on the same day for the signs and symptoms of inflammation and total leucocyte count. Two endpoints were considered in the management. First was the absence of pus on aspiration, and second, the absence of residual abscess on ultrasonography. Intravenous or oral cloxacillin 500 mg (four times a day) was given empirically to all the patients, and antibiotics were changed according to the microbiology report. A total of 10 days of antibiotics was given to all patients. Patients with lactational breast abscesses were encouraged to breastfeed from both breasts. In group A, if there were signs and symptoms of inflammation after the two aspirations or raised leucocyte count (>11000 cells/mm^3^) or ultrasonography showing residual abscess (assessed on the seventh day), it was considered to be a failure of treatment, and were taken up for ID.

Group B patients underwent ID under general anesthesia on an inpatient basis after an initial USG of the breast. The drained collection was sent for culture and sensitivity tests. Intravenous or oral cloxacillin 500 mg (four times a day) was given empirically on the day of surgery and was changed to sensitive antibiotics per pus culture and sensitivity report. The patients were discharged on the third day of surgical drainage. Patients were advised for daily dressing from the outpatient department. Clinical examination was done to look for purulent discharge and signs of inflammation on the first, third, fifth and seventh days. The total leucocyte count was done on the first, third, fifth and seventh days. The antibiotic was given for 10 days to all the patients. All patients with lactational breast abscesses were encouraged to breastfeed from opposite sides with the milk expression on the same side. In group B, if there were signs of inflammation, raised leucocyte count, or persistent discharge from the surgical site even after the seventh day, it was considered a treatment failure. Such patients were taken for re-exploration of breast abscess.

In both groups, the intervention outcome was recorded and analyzed as postoperative pain, time to heal, residual abscess or recurrence, fistula formation, the appearance of scarring, and failure of treatment. On the day of normalization of the leucocyte count (<11000 cells/mm^3^), absent purulent discharge and no signs of inflammation in group A, no clinical signs of inflammation, ultrasonography did not show collection, normalized leukocyte count (<11000 cells/mm^3^) in group B was considered as the day of start of healing, and healing time (days) was documented. Visual analog scale (VAS) score measured postoperative pain during the immediate post-intervention period, 24 hours after the intervention, and 48 hours after the intervention. If VAS >4, then analgesics were given [9].

## Results

Out of the 46 patients who sought care at the surgical OPD and emergency, a total of 44 patients who met the specified inclusion criteria were enrolled in the study. Of the 44 patients, 23 (52%) in group A and 21 (48%) in group B were analyzed. Out of 44 BA patients, 23 patients (52.27%) had BA in the right breast, 19 patients (43.18%) had BA in the left breast, and only two patients (4.54%) had it in both breasts. The right upper outer quadrant was most frequently affected (24.52%), followed by the left upper outer quadrant (16.98%). We compared the various characteristics of the patients with BA between group A and group B. These include age, complaints (duration of the symptoms, presence of pain, lump, fever, history of similar episodes), lactational history, signs (tachycardia), USG features of the BA (size), and site of BA (right, left or bilateral). However, none of these factors were significant, as the p-value for all these factors was more than 0.05 (Table 1).

**Table 1:** Distribution of baseline characteristics between two groups. SD: standard deviation; USG: ultrasonography

Characteristics	Group A (N=23)	Group B (N=21)	p-value
Age in years (mean ± SD)	30.34 ± 9.53	26.33 ± 7.16	0.125
Duration of symptoms (days)	6.35 ± 3.15	5.48 ± 2.80	0.340
Pain	21	20	1.0
Lump	19	21	0.109
Fever	3	9	0.027
Similar past episode	5	4	1.00
Tachycardia	9	12	0.232
Size (cm) of abscess under USG (<3 cm)	6	2	0.36
Size (cm) of abscess under USG (3 cm to 5 cm)	7	7	0.36
Size (cm) of abscess under USG (>5 cm)	10	12	0.36
Right breast abscess	14	9	0.11
Left breast abscess	7	12	0.11
Bilateral breast abscess	2	0	0.11
Lactational	14	16	0.27
Non Lactational	9	5	0.27

We compared the presence of signs of inflammation and pus discharge between both groups on day one, day three, day five, and day seven post-intervention. However, this was not found to be statistically significant (p-value >0.05). We conducted a comparison of the total leucocyte count between the groups following the intervention, specifically on day one, day three, day five, and day seven. Following our thorough analysis, we did not uncover any statistically significant differences, as all calculated p-values exceeded the threshold of 0.05, indicating a lack of significant divergence among the measured variables (Table 2).

**Table 2:** Comparison of response to intervention in patients with breast abscess.

Signs of inflammation/pus discharge	Group A (N=23)	Group B (N=21)	p-value	Leucocyte count (cells/mm^3^)	Group A (N=23)	Group B (N=21)	p-value
Day 1	22	21	1.000	Day 1	11	15	0.112
Day 3	20	19	1.000	Day 3	8	8	0.820
Day 5	11	10	0.989	Day 5	7	5	0.622
Day 7	6	5	0.862	Day 7	6	5	0.862

We compared the post-intervention outcomes in both groups. Six patients in group A and five patients in group B had treatment failure (defined in the methodology section). The p-value was found to be 0.862 and was statistically not significant. The mean duration of the clinical resolution of the BA in group A was 5.12 ± 1.31 days, and in group B was 8.14 ± 3.75 days. The p-value was found to be 0.002 and statistically significant. In group B, 13 patients had ceased breastfeeding, whereas in group A, only four patients had done so, and this disparity was found to be statistically significant with a p-value of 0.005. The healing period (in days) was compared between both groups. In group A, it was 44.8 ± 3.41 days, and in group B, it was 35.56 ± 1.59 days and was statistically significant (p-value <0.001). Scar formation was assessed following the intervention in both groups, and the results indicated a highly significant difference with a p-value of <0.0001. We also compared formation and recurrence in both the groups post-intervention; however, they were not statistically significant, with p-values of 1.0 and 1.0 respectively (Table 3).

**Table 3:** Comparison of the outcomes (post-intervention) in both groups.

Outcomes (post-intervention)	Group A (N=23)	Group B (N=21)	p-value
Treatment failure	6	5	0.862
Clinical resolution of the abscess (days) (mean)	5.12 ± 1.31	8.14 ± 3.75	0.002
Discontinuation of breastfeeding	4	13	0.005
Healing period (days)	35.56 ± 1.59	44.8 ± 3.41	<0.001
Fistula formation	1	1	1.000
Scar formation	0	16	<0.0001
Recurrence	2	2	1.000

We analysed the growth of various organisms in the abscess by sending the culture of the pus obtained. In both groups, Staphylococcus aureus was the most common organism gown. Fourteen patients in group A and eight patients in group B had growth of Staphylococcus aureus. Few patients had growth of gram-negative organisms and few patients did not show any growth (two patients in group A and three patients in group B) (Table 4).

**Table 4:** Growth of organisms in both groups. MRSA: methicillin resistant Staphylococcus aureus

Culture organism	Group A (N=23)	Group B (N=21)
Staphylococcus aureus (including MRSA)	14	8
Klebsiella pneumonia	0	2
Citrobacter koseri	4	1
Burkholderia contaminans	1	0
Enterococcus faecalis	1	1
Proteus vulgaris	0	2
Morganella morganii	0	2
Streptococcus mitis	1	0
Streptococcus pyogenes	0	2
No growth	2	3

**Figure 2:**
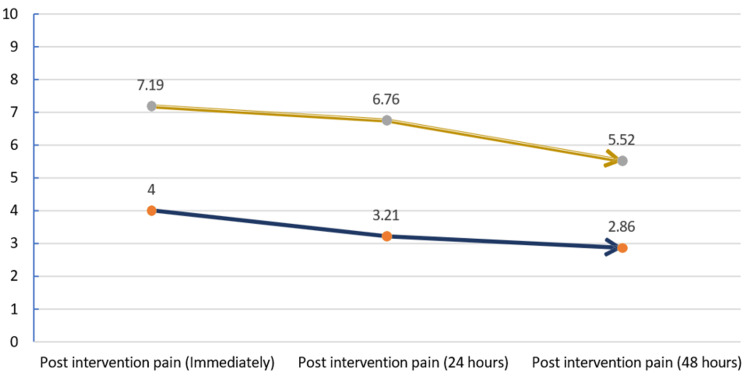
Comparison of postoperative pain by visual analogue scale (VAS) between the groups.

We analyzed pain (post-intervention) immediately after the procedure, 24 hours, and 48 hours after the intervention, with the help of a VAS score. In group A and group B, immediate post-intervention pain assessment, the mean (SD) VAS score was 4.00 (±0.79) and 7.19 (±0.87), respectively. After 24 hours of intervention, the mean (SD) of postintervention pain was 3.21 (±0.99) and 6.76 (±0.99), respectively. The last post-intervention pain assessment was done after 48 hours, and the mean (SD) of post-intervention pain between group A and group B were 2.86 (±1.14) and 5.52 (±1.24), respectively. Significant differences in the means (standard deviation) of post-intervention pain scores were observed between groups A and group B, with a p-value of <0.001 (Figure 2).

## Discussion

BA is a common condition in females, especially during the lactational period. The treatment of BA can be done by traditional incision and drainage or by USG-guided needle aspiration. Nonetheless, a Cochrane Systematic Review in 2015, focused on the treatment of breast abscesses, concluded that there is inadequate evidence to establish the superiority of needle aspiration over incision and drainage in the management of breast abscesses [1]. The present study was a randomized controlled non-inferiority trial comparing the outcomes and effectiveness of USG-guided needle aspiration and conventional ID for treating BA.

BA can be treated by conventional ID. However, there is a rising trend toward employing minimally invasive procedures [1]. The needle aspiration is considered to be an alternative and less invasive approach for the treatment of BA [10]. The Society of Interventional Radiology (SIR) defines image‐guided percutaneous aspiration as "evacuation or diagnostic sampling of a fluid collection with the use of a catheter or a needle during a single imaging session, with the removal of the catheter or needle immediately after the aspiration" [11]. World Health Organization (WHO) supports using USG-guided aspiration of the lactational BA [12]. While needle aspiration is generally regarded as a less invasive approach, it's important to note that not all cases of breast abscesses can be effectively treated with needle aspiration, and ID may be necessary. Needle aspirations may be done as a single-stage procedure or with multiple sessions. In cases where the aspirate exhibits a viscous consistency, the addition of a saline or antibiotic solution can aid in facilitating the aspiration process [1].

Recent studies have supported the use of USG-guided aspiration of the BA, as it has multiple advantages. These include less scarring, a continuation of breastfeeding, and less expensive than the conventional ID as it does not need hospitalization and anesthesia [10,13]. However, multiple aspirations are often required. High failure rates have been noted with multiloculated abscesses and cavity sizes of more than 5 cm [6,8]. Patients with failed attempts of repeated aspirations end up requiring incision and drainage to resolve BA. However, there are no guidelines for USG-guided needle aspiration of the BA. Our study was conducted to evaluate the outcomes and effectiveness of USG-guided needle aspiration to that of conventional ID in patients having BA. 

We compared age, presenting symptoms, tachycardia, abscess size, site, and lactation in both groups. The most common symptom of the BA in our study was pain and a lump in the breast. We also compared the signs of inflammation or pus discharge and total leucocyte count in both the groups on post-intervention day one, day three, day five, and day seven. However, none of these were statistically significant. We compared the various outcomes following intervention in both groups. There was a higher number of patients with treatment failure in group A. However, this was not statistically significant (p-value=0.862). In our study, the clinical resolution of the abscess was early group A, and it was statistically significant (p-value=0.002). The results of the previous studies are varied compared to the present study. Many studies have shown that USG-guided drainage was successful in the majority of the patients, whereas one study showed a higher failure rate in the USG-guided drainage of BA [14-18]. In our study, this higher failure rate may be due to the inclusion of patients with BA more than 5 cm, compared to other studies.

Almost four (17.4%) patients had discontinued breastfeeding in group A, and 13 (61.9%) patients had discontinued breastfeeding in group B. This was statistically significant (p-value=0.005). Colin et al., in their retrospective study, showed that 91% of patients with BA continued breastfeeding in the ultrasound-guided percutaneous drainage group [7]. Three other studies analyzed the continuation of breastfeeding during nonsurgical management of abscesses with mixed results [19-21]. We conducted a comparison of the healing time between both groups. Group A exhibited a significantly shorter healing time in contrast to the other groups, with a highly significant p-value of <0.001. Other studies by Naeem et al. and Eryilmaz et al. also showed similar results to our study, with healing time being statistically significant [14,22]. This is because of the apparent reason that the surgical wound needs more time to heal.

We compared fistula formation post-intervention and recurrence in both groups. In our study, one patient in each group developed a fistula, and two had recurrence. However, these factors were not statistically significant. A study by Christensen et al. showed that only four of the 151 patients who received USG-guided drainage developed a fistula [23]. We compared scar formation in both groups. As ID is an operative procedure and hence had high rates of scar formation, it was statistically significant (p-value <0.0001). The main culprit responsible for breast abscesses is Staphylococcus aureus. The microbiology of this condition is complex. In the past, methicillin-resistant Staphylococcus aureus (MRSA) was primarily associated with healthcare-associated infections. However, in recent times, it has emerged as a pathogen involved in a spectrum of community-acquired infections, including breast abscesses [23].

Our study also analyzed postoperative pain response (VAS score) immediately after the intervention, 24 hours after the intervention, and 48 hours after the intervention. We found statistically significant differences in all three assessment periods. Odiya et al. also reported similar findings; they compared pain severity between incision and drainage versus minimally invasive percutaneous placement of a suction drain [24]. As they gave analgesics if the VAS score was more than 4, patients of the intervention group complained of less severe pain. They required less duration of analgesics than the control group. 

There are a few limitations of the present study. Blinding was not performed in this study, which may affect participants’ behavior and reactions to subjective outcome measures like pain. The study could not reach a predetermined, calculated sample size due to the coronavirus disease 2019 (COVID-19) pandemic. For this reason, the study could not prove the non-inferiority of USG-guided needle aspiration over surgical drainage. Few patients were managed by teleconsultation during the COVID-19 pandemic. USG is a highly operator-dependent radiological intervention. The experience of a sociologist has an immense effect on intervention capability and completeness. We did not evaluate the cost-benefit analysis for the USG-guided needle aspiration for BA. 

## Conclusions

From the present study, the minimally invasive USG-guided needle aspiration of BA is a safe technique. It avoids the need for surgical procedures anesthesia, a significant decrease in post-intervention pain, and hospital admission. USG-guided needle aspiration will not have scar formation, and the patient can continue breastfeeding. Hence, patients and surgeons should be encouraged to use minimally invasive needle aspiration for BA unless contraindicated. However, further studies will be required with large sample sizes for conclusive evidence and to form proper guidelines. 
